# Molecular Mechanisms in Oral Squamous Cell Carcinoma: Integrative Roles of Cancer-Associated Fibroblasts, Immune Microenvironment, and Precision Therapeutic Opportunities

**DOI:** 10.3390/ijms27072956

**Published:** 2026-03-24

**Authors:** Chung-Che Tsai, Po-Chih Hsu, Chan-Yen Kuo

**Affiliations:** 1 Department of Dentistry, Taipei Tzu Chi Hospital, Buddhist Tzu Chi Medical Foundation, New Taipei City 231, Taiwan; chungche.tsai@gmail.com; 2 Institute of Oral Medicine and Materials, College of Medicine, Tzu Chi University, Hualien 970, Taiwan

**Keywords:** oral squamous cell carcinoma, tumor microenvironment, cancer-associated fibroblasts, immune regulation, epigenetics, metabolic reprogramming, precision oncology

## Abstract

Oral squamous cell carcinoma (OSCC) remains a major global health burden due to aggressive invasion, early metastasis, therapeutic resistance, and poor long-term survival. Beyond tumor-intrinsic genetic and epigenetic alterations, accumulating evidence highlights the critical role of the tumor microenvironment in shaping OSCC progression and clinical outcomes. Cancer-associated fibroblasts (CAFs) and immune cells orchestrate tumor initiation, immune evasion, and recurrence through extracellular matrix remodeling, cytokine signaling, angiogenesis, and metabolic and redox regulation. Key oncogenic pathways, including EGFR/PI3K/AKT/mTOR, TGF-β, Wnt, and Notch, integrate with non-coding RNA networks to reinforce stemness, epithelial–mesenchymal transition, and therapy resistance. Moreover, PD-1/PD-L1-mediated immune escape, CAF-driven biomechanical remodeling, and metabolic reprogramming such as aerobic glycolysis and lipid metabolism contribute to OSCC heterogeneity. This review synthesizes current insights into OSCC across genomic, epigenetic, metabolic, and microenvironmental dimensions, emphasizing CAF biology, immune landscape reprogramming, and non-coding RNA regulation. We further discuss emerging biomarkers, liquid biopsy approaches, and targeted therapeutic strategies, providing a system-level framework for biomarker-guided stratification and precision combination therapies in OSCC.

## 1. Introduction

Oral malignancies constitute a major subset of head and neck cancers, among which oral squamous cell carcinoma (OSCC) predominates, representing the vast majority of diagnosed cases [1]. From an epidemiological perspective, the occurrence of OSCC varies substantially across regions, with notably higher rates reported in South and Southeast Asia [2]. This distribution is closely associated with prevalent lifestyle and environmental exposures, including betel quid chewing, tobacco smoking, and alcohol intake [3]. Other recognized risk factors encompass chronic oral inflammation, inadequate oral hygiene, and infection with oncogenic viruses such as human papillomavirus (HPV) [4]. Clinically, OSCC continues to be associated with unfavorable outcomes, with long-term survival showing little improvement over time. The persistently modest five-year survival rates are largely attributable to the frequent diagnosis of disease at advanced stages [5]. Standard therapeutic approaches can achieve satisfactory control of primary lesions, yet they frequently prove insufficient in suppressing disease relapse and distant spread [6]. These challenges underscore gaps in our current knowledge of the molecular and cellular processes that drive OSCC development, disease advancement, and resistance to treatment [7].

**Figure 1:**
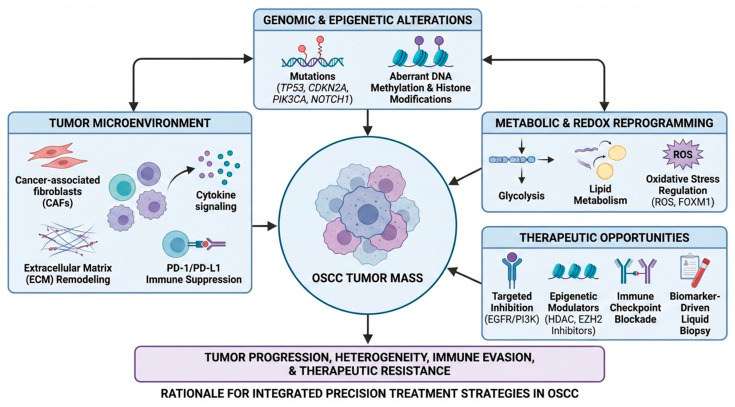
System-level framework of OSCC progression. This conceptual diagram illustrates the hierarchical and dynamic interactions between tumor-intrinsic molecular alterations and tumor microenvironmental networks in OSCC. At the tumor-intrinsic level, genomic alterations and epigenetic reprogramming activate oncogenic signaling pathways that promote tumor proliferation, survival, and metabolic adaptation. These molecular events influence the surrounding tumor microenvironment by regulating immune cell recruitment, CAF activation, and extracellular matrix remodeling. In turn, stromal and immune components generate reciprocal signals that further modulate tumor signaling networks, metabolic plasticity, and therapeutic responses. Together, these interconnected processes form a system-level regulatory network that drives tumor heterogeneity, disease progression, and treatment resistance while revealing potential biomarkers and therapeutic targets relevant to precision oncology strategies in OSCC. Single-headed arrow indicates a unidirectional effect or regulatory influence, whereas double-headed arrow represents bidirectional or reciprocal interactions between components.

Recent methodological advances, including high-throughput sequencing, single-cell analysis, spatial profiling, and integrative systems approaches, have demonstrated that OSCC extends beyond abnormalities confined to malignant epithelial cells [8]. Rather than being driven solely by malignant cells, OSCC is now understood as a multifaceted system characterized by continuous crosstalk between tumor cells and surrounding stromal fibroblasts, immune populations, endothelial cells, and the extracellular matrix (ECM) [9]. Notably, stromal fibroblasts with cancer-associated phenotypes and infiltrating immune cells exert a dominant influence on tumor growth and progression [10]. Rather than representing independent biological layers, these molecular and microenvironmental processes operate as an interconnected regulatory network that drives OSCC evolution. Genomic alterations establish oncogenic signaling programs, while epigenetic reprogramming and non-coding RNA networks reshape transcriptional states that support tumor plasticity. In parallel, cancer-associated fibroblasts and immune cells remodel the tumor microenvironment through cytokine signaling, extracellular matrix dynamics, and immune checkpoint regulation. Metabolic and redox adaptations further reinforce these processes by enabling tumor cells to survive therapeutic stress. Conceptually, OSCC progression can be viewed as a system-level disease driven by dynamic interactions between tumor-intrinsic molecular alterations and tumor microenvironmental networks, which together shape tumor heterogeneity, therapeutic resistance, and clinical outcomes. This conceptual framework provides the foundation for the integrative analysis presented throughout this review. Accordingly, this review synthesizes emerging molecular insights into OSCC pathogenesis, emphasizing cancer-associated fibroblast (CAF) biology and immune microenvironment remodeling as central drivers of disease progression and precision therapeutic opportunities. The system-level framework of OSCC pathogenesis discussed in this review is conceptually illustrated in Figure 1, highlighting the dynamic and hierarchical interactions between tumor-intrinsic molecular alterations and tumor microenvironmental networks, including stromal, immune, and metabolic components. Table 1 further summarizes the major molecular and microenvironmental processes involved in OSCC progression and highlights their potential diagnostic biomarkers, therapeutic targets, and implications for precision oncology strategies.

## 2. Genomic Landscape of Oral Cancers

### 2.1. Key Oncogenic and Tumor Suppressor Pathways Shaping the Molecular Landscape and Clinical Behavior of OSCC

Large-scale genomic analyses, most notably those conducted through The Cancer Genome Atlas (TCGA), have uncovered recurrent somatic alterations that underpin the molecular basis of OSCC [11]. Tumor protein p53 (*TP53*) mutations occur in over 70% of cases and represent a hallmark of genomic instability [12]. Disruption of *TP53* activity weakens cellular surveillance mechanisms, including genomic damage sensing, programmed cell death, and cell-cycle regulation, thereby promoting oncogenic transformation [13]. Alterations in the *TP53* gene represent one of the most frequent and biologically significant molecular events in OSCC, contributing to dysregulated cell-cycle control, genomic instability, and tumor progression. Consequently, monitoring *TP53* mutational status has been proposed as a potential diagnostic and prognostic biomarker in OSCC; however, its clinical utility remains context-dependent and varies according to mutation subtype, HPV status, and disease stage [14]. It should be noted that *TP53* mutational status and TP53 protein expression are not interchangeable parameters, and their prognostic implications may differ.

In OSCC patients with pathological lymph node metastasis, elevated cyclin-dependent kinase inhibitor 2A (*CDKN2A*) expression, which encodes p16 protein, is significantly associated with a higher risk of distant metastasis and reduced overall survival, whereas *TP53* expression shows no prognostic significance [15]. Wang et al. demonstrated that higher *CDKN2A* expression is independently associated with improved overall survival in head and neck squamous cell carcinoma (HNSCC) and that an AI-driven pathomics model can reliably infer *CDKN2A* status from routine histopathology images [16]. Nałecz et al. showed that p16 and Ki-67 protein levels vary across HNSCC subtypes and are associated with nodal status, HPV infection, and environmental factors such as alcohol consumption and smoking, reflecting heterogeneity in tumor biology [17]. In recurrent or metastatic HNSCC, functional inactivation of *CDKN2A* is linked to inferior survival outcomes, even in the setting of immune checkpoint therapy [18]. Taken together, current evidence suggests that *CDKN2A* status provides meaningful prognostic insight in OSCC and HNSCC, with its clinical impact varying according to disease stage, biological context, and therapeutic setting. By comparison, *TP53* expression offers limited prognostic value, underscoring the potential utility of *CDKN2A* as a more informative marker for patient stratification and individualized management.

*NOTCH1* signaling in HNSCC consistently functions as a tumor-suppressive pathway by promoting very early squamous differentiation, suppressing oncogenic programs, and reducing tumor-initiating cell frequency, regardless of mutational status [19]. Moreover, aberrant activation of *NOTCH1* signaling is closely associated with aggressive clinicopathological features and independently predicts poor disease-specific survival in patients with OSCC [20]. A previous study indicated that genetic alterations in *NOTCH1* occur frequently in OSCC and are linked to disrupted Notch signaling, as evidenced by changes in NOTCH1 intracellular domain expression [21]. Regarding the oncogenic role of *NOTCH1* in OSCC progression, elevated cytoplasmic NOTCH1 expression is frequently observed in OSCC and is particularly enriched in moderately to poorly differentiated tumors, where it correlates with significantly reduced overall survival [22]. Another study showed that an early shift toward Notch1-Delta-like-4 (NOTCH1-Dll4) signaling is preserved during OSCC progression, supporting a poorly differentiated cellular state and enhanced invasiveness [23]. Altogether, the evidence suggests that *NOTCH1* signaling in HNSCC/OSCC plays a dual and stage-specific role, restraining tumor initiation through early differentiation while promoting malignant behavior when improperly activated. Persistent NOTCH1-Dll4 activity is linked to dedifferentiation, increased invasiveness, and unfavorable prognosis, highlighting its relevance for risk stratification and targeted intervention.

Phosphatidylinositol-4,5-bisphosphate 3-kinase catalytic subunit alpha (*PIK3CA*), which encodes the p110α catalytic subunit of phosphatidylinositol 3-kinase (PI3K), is one of the most frequently altered oncogenes in OSCC [24]. These genetic alterations correlate with increased phosphorylation of downstream protein kinase B (AKT) signaling components and are linked to augmented tumor cell proliferation, survival signaling, metabolic pathway activation, and reduced apoptotic responses in OSCC tissues and experimental models, supporting a direct role of aberrant *PIK3CA*-mediated activation of the PI3K/AKT/mTOR signaling axis, a central oncogenic pathway in OSCC (see Section 5.1) [25]. A study demonstrated that *PIK3CA* hotspot mutations can be reliably identified in OSCC tumor tissue and are frequently detectable in matched saliva samples, supporting the feasibility of saliva-based liquid biopsy [26]. *PIK3CA* mutations were detected in a subset of oral cavity (OC)-SCC cases at a frequency comparable to other populations, with alterations in exon 20 being predominant and showing site-specific associations, particularly in tongue tumors [27]. This work shows that the synergistic activation of *PIK3CA* signaling and carcinogen exposure markedly hastens oral tumor development and recapitulates key pathological and biological characteristics of human OSCC. Accordingly, a *PIK3CA* transgenic mouse with a 4-nitroquinoline 1-oxide (4NQO) treatment model provides a valuable experimental system for studying disease mechanisms and evaluating translational approaches in OSCC research [33]. Starzyńska et al. indicate that loss of PTEN protein expression and *PIK3CA* gene amplification are recurrent events in OSCC and are associated with adverse clinicopathological features and survival outcomes [34]. Overall, accumulated evidence indicates that aberrations in *PIK3CA* play a pivotal role in OSCC by sustaining PI3K/AKT signaling, promoting malignant progression, and correlating with unfavorable pathological features and prognosis. The consistent identification of *PIK3CA* alterations across patient samples, liquid biopsy approaches, and experimental models further supports its clinical relevance as a target for diagnosis and therapeutic intervention in OSCC.

Accumulating evidence indicates that Harvey Rat sarcoma viral oncogene homolog (*HRAS*) mutations contribute to OSCC by sustaining the aberrant activation of key signaling pathways, particularly the RAS-mitogen-activated protein kinase (MAPK) cascade, thereby promoting dysregulated cell proliferation and survival. However, population-specific mutation patterns and unresolved mechanisms involving post-translational modifications underscore the need for further molecular studies to clarify the prognostic and therapeutic relevance of *HRAS* in OSCC [28]. Accumulating clinical and genomic evidence demonstrates that alterations activating the MAPK pathway define a biologically distinct subset of HNSCC with actionable therapeutic vulnerabilities [29]. Hoxhallari et al. reported that elevated expression of wild-type *HRAS* in HNSCC is linked to sustained activation of extracellular signal-regulated kinase (ERK) and c-Jun signaling, leading to increased expression of key nucleotide excision repair components such as excision repair cross-complementation group 1 (*ERCC1*) and DNA polymerase delta 4, accessory subunit (*POLD4*). This molecular profile is associated with an enhanced DNA repair response that correlates with diminished platinum responsiveness [30]. The systematic review by Devi et al. shows that aberrant *HRAS* expression and recurrent hotspot mutations, particularly at codons 12, 13, and 61, are consistently observed in OSCC and are associated with specific clinicopathological features and risk behaviors such as tobacco exposure [31]. Squamous cell papillomas of the head and neck are characterized predominantly by mutually exclusive activation of the RAS-MAPK pathway through either *KRAS*/*HRAS* mutations or low-risk HPV infection, with distinct anatomic and clinicopathological associations. The divergent mutational spectrum from HNSCC supports the notion that squamous cell papilloma represents a biologically benign lesion rather than a precursor to malignancy [32]. Altogether, these findings indicate that current molecular and clinical evidence indicates that *HRAS*-driven MAPK pathway activation plays a context-dependent role across head and neck lesions, contributing to tumor biology, treatment response, and molecular heterogeneity in OSCC. In contrast, the mutually exclusive involvement of *HRAS*/*KRAS* alterations or low-risk HPV infection in squamous cell papilloma highlights a distinct, predominantly benign molecular framework that is biologically separate from malignant HNSCC.

### 2.2. Copy Number Alterations and Structural Variants

In addition to recurrent point mutations, OSCC is characterized by extensive copy number alterations (CNAs) across the genome [35]. High-level copy number amplifications affecting oncogenic loci such as epidermal growth factor receptor (*EGFR*), cyclin D1 (*CCND1*), MYC proto-oncogene, bHLH transcription factor (*MYC*), and SRY-box transcription factor 2 (*SOX2*) are recurrent genomic events in OSCC, as demonstrated by large-scale genomic profiling studies on OSCC and HNSCC cohorts [36]. The amplification of *EGFR* and *CCND1* is consistently associated with enhanced proliferative signaling and cell-cycle regulation disruption [37], while copy number gains involving *MYC* contribute to transcriptional programs that support sustained tumor cell growth [38]. In parallel, recurrent amplification of chromosome 3q regions encompassing *SOX2* has been linked to the maintenance of stem-like transcriptional programs in OSCC [39]. Overall, extensive copy number alterations represent a central genomic feature of OSCC, with recurrent amplifications of *EGFR*, *CCND1*, *MYC*, and *SOX2* converging on proliferative signaling, cell-cycle dysregulation, and stemness-associated transcriptional programs. These coordinated genomic gains underscore the importance of CNAs as key drivers of OSCC progression and biological heterogeneity.

### 2.3. Oncogenic Copy Number Amplifications as Key Determinants of OSCC Progression and Biological Diversity

Intratumoral heterogeneity is increasingly recognized as a defining biological feature of OSCC, arising from ongoing genomic instability and Darwinian clonal evolution within the same lesion [40,41]. Consequently, spatially and temporally distinct subclones can coexist, exhibiting divergent mutational spectra, transcriptional programs, and microenvironmental interactions, features that are now being resolved with multi-regional sampling, single-cell profiling, and spatial transcriptomic approaches in OSCC/HNSCC [42]. Under therapeutic pressure, including surgery plus adjuvant radiotherapy/chemotherapy, targeted therapy, or immunotherapy, treatment-sensitive populations may be depleted while pre-existing or adaptively reprogrammed resistant clones expand, ultimately seeding minimal residual disease and clinical relapse [43]. In parallel, heterogeneity within malignant compartments and the surrounding tumor microenvironment can shape differential drug exposure, immune surveillance, and stress-adaptive states, including partial epithelial–mesenchymal transition (EMT)-like programs, further compounding variable treatment response across tumor regions and over time [44,45]. In summary, this evolutionary framework supports the need for integrative and adaptive therapeutic strategies in OSCC, such as biomarker-guided combination regimens, longitudinal molecular monitoring, and approaches that account for spatially segregated resistant niches to mitigate clonal selection and reduce the risk of recurrence.

## 3. Epigenetic Reprogramming in Oral Carcinogenesis

### 3.1. Aberrant DNA Methylation Orchestrates Tumor Suppressor Silencing and Malignant Progression in OSCC

Epigenetic dysregulation plays a critical role in OSCC progression [46]. In OSCC, global DNA hypomethylation has been frequently observed and is associated with chromosomal instability, which may facilitate aberrant chromosomal recombination and replication stress, leading to the accumulation of copy number alterations [47]. These epigenetic changes are thought to increase genomic plasticity during OSCC progression and clonal evolution, thereby contributing to tumor heterogeneity and disease advancement [7]. Moreover, promoter-specific hypermethylation is a recurrent epigenetic alteration that is consistently associated with transcriptional silencing of tumor suppressor genes, including cadherin 1 (*CDH1*), *CDKN2A*, and Ras association domain family member 1, isoform A (*RASSF1A*) [48,49,50]. Aberrant methylation of the *CDH1* promoter has been correlated with reduced E-cadherin expression and compromised cell–cell adhesion in OSCC tissues [51]. Likewise, promoter hypermethylation of *CDKN2A* is frequently observed in OSCC and is associated with loss of p16 expression and deregulation of cell-cycle control [52]. In addition, epigenetic inactivation of *RASSF1A* through promoter hypermethylation has been reported in OSCC and is linked to alterations in apoptosis-related signaling pathways [53]. Thus, these findings indicate that promoter-specific DNA hypermethylation represents a key epigenetic feature of OSCC, contributing to malignant progression through coordinated silencing of multiple tumor suppressor genes.

### 3.2. Epigenetic Reprogramming Through Dysregulated Histone Acetylation and Methylation in OSCC: Roles of Histone Deacetylases and Enhancer of Zeste Homolog 2 as Therapeutic Targets

Alterations in histone acetylation and methylation constitute a major epigenetic mechanism underlying transcriptional reprogramming in OSCC [54]. Dysregulated histone acetylation has been frequently reported in OSCC and is closely associated with aberrant chromatin compaction and altered accessibility of gene regulatory regions [55]. In particular, overexpression of histone deacetylases (HDACs) has been consistently observed in OSCC tissues and is correlated with unfavorable clinicopathological features, including advanced tumor stage and poor differentiation [56]. Increased HDAC activity is associated with reduced acetylation of histone H3 and H4 tails, which in turn corresponds to transcriptional repression of genes involved in cell-cycle regulation, differentiation, and apoptosis [57].

Concurrently, aberrant histone methylation patterns have been documented in OSCC or HNSCC, most notably through the upregulation of enhancer of zeste homolog 2 (EZH2), the catalytic subunit of the polycomb repressive complex 2 (PRC2) [58,59]. Elevated EZH2 expression in esophageal squamous cell carcinoma has been associated with increased trimethylation of histone H3 at lysine 27 (H3K27me3), a repressive chromatin mark linked to the silencing of tumor suppressor and differentiation-related genes [60,61]. Clinical and molecular studies have demonstrated that high EZH2 expression correlates with aggressive tumor behavior, lymph node metastasis, and adverse patient outcomes in OSCC or HNSCC cohorts [42,62,63]. Collectively, these findings indicate that dysregulated histone-modifying enzymes contribute to altered chromatin architecture and transcriptional control in OSCC. Given that histone acetylation and methylation are dynamically regulated and reversible epigenetic processes, HDACs and EZH2 have been extensively investigated as therapeutic targets in preclinical and clinical studies of OSCC. These epigenetic alterations highlight potential opportunities for therapeutic intervention using epigenetic modulators such as HDAC or EZH2 inhibitors.

## 4. Non-Coding RNAs as Regulatory Hubs

### 4.1. MicroRNA Dysregulation in OSCC: Diagnostic, Prognostic, and Therapeutic Implications Across Molecular Pathways

A previous review summarized how dysregulated microRNAs modulate the Akt/mechanistic target of rapamycin (mTOR) signaling pathway in OSCC, thereby promoting tumor proliferation, survival, invasion, and therapy resistance. It also highlighted emerging microRNA (miRNA)-based therapeutic strategies targeting this pathway as potential approaches to improve OSCC treatment outcomes [64]. Another review integrated genomic and epigenetic data to identify key regulatory miRNAs in OSCC, highlighting miR-34a-5p, miR-155-5p, miR-124-3p, miR-1-3p, and miR-16-5p as disease-relevant regulators of major oncogenes and tumor suppressors. It comprehensively summarized their dysregulated expression patterns, validated targets, and downstream signaling pathways [65]. miRNA profiling studies have suggested that miR-21 is consistently upregulated and miR-145 is downregulated in oral leukoplakia, indicating their potential utility as diagnostic and prognostic biomarkers for malignant transformation [66]. miR-31 is consistently upregulated in the plasma, saliva, and tumor tissue of patients with OSCC, where it functions predominantly as an oncogenic regulator by modulating multiple target genes and signaling pathways involved in tumor initiation, progression, and metastasis [67]. A study identified 13 upregulated miRNAs in tongue OSCC using TCGA data, with 5 miRNAs, particularly miR-196b, demonstrating strong diagnostic performance in distinguishing cancerous from normal tissues [68]. Rajan et al. delineated a prognostic miRNA signature in OSCC, demonstrating that coordinated upregulation of miR-196a and suppression of miR-204, specifically in terms of their combined expression ratio, serves as a robust indicator of tumor aggressiveness, recurrence risk, and patient survival outcomes [69]. A previous scoping review synthesized evidence from 54 eligible studies and showed that multiple dysregulated miRNAs exert experimentally validated regulatory effects on OSCC proliferation, invasion, and metastasis, thereby supporting the concept that therapeutic restoration of tumor-suppressive miRNA expression may constitute a viable anticancer strategy, pending further advances in delivery technologies and clinical translation [70]. Moreover, salivary cytokines and miRNAs—most notably interleukin-6 (IL-6), tumor necrosis factor-α (TNF-α), and matrix metalloproteinase-9 (MMP-9)—exhibit characteristic alterations in patients with OSCC and may function as accessible, non-invasive indicators for both early detection and prognostic evaluation [71]. Overall, the data have increasingly supported the promise of miRNA-based strategies for improving early detection and treatment outcomes in OSCC, although further clinical validation remains necessary. Collectively, these findings suggest that non-coding RNA networks may represent promising diagnostic biomarkers and potential therapeutic targets in OSCC.

### 4.2. Dysregulated lncRNA- and circRNA-Mediated Regulatory Networks Driving Epigenetic Reprogramming and Therapeutic Resistance in OSCC

Long non-coding RNAs (lncRNAs) and circular RNAs (circRNAs) orchestrate gene-expression programs through multiple layers of regulation, acting not only as competitive endogenous RNAs (ceRNAs) that sequester miRNAs but also as scaffolds that recruit chromatin-modifying complexes and transcriptional regulators to specific loci, thereby reshaping epigenetic states and downstream transcriptional outputs [72,73,74]. In OSCC, dysregulated lncRNAs have been repeatedly linked to aggressive phenotypes by coordinating stemness-associated signaling, metabolic adaptation, and therapeutic tolerance [75,76]. For example, metastasis-associated lung adenocarcinoma transcript 1 (*MALAT1*) upregulation in OSCC or HNSCC has been shown to enhance oncogenic programs through a ceRNA axis involving miR-101 and the PRC2 catalytic subunit EZH2, supporting a mechanistic connection between lncRNA deregulation and chromatin-mediated transcriptional repression [77,78].

Beyond its established roles in tumor growth and invasion, *MALAT1* has also been implicated in the acquisition of cisplatin resistance in OSCC [79]. Experimental studies using cisplatin-resistant OSCC cell models have demonstrated that *MALAT1* upregulation is associated with enhanced activation of the PI3K/AKT/mTOR signaling axis (see Section 5.1), a key survival pathway linked to chemotherapy tolerance [80]. In parallel, *MALAT1* overexpression correlates with EMT-associated phenotypic changes, including reduced epithelial marker expression and increased mesenchymal traits [81], as well as the upregulation of multidrug resistance-related features such as P-glycoprotein expression [82]. Furthermore, observations derived from cisplatin-resistant OSCC experimental models support an association between *MALAT1* dysregulation and signaling as well as phenotypic adaptations commonly observed in cisplatin resistance.

HOX transcript antisense RNA (*HOTAIR*), a well-characterized chromatin-associated long non-coding RNA involved in epigenetic regulation, has been reported in several studies to be associated with therapeutic resistance in oral malignancies [83,84,85]. Zhang et al. indicated that *HOTAIR*-associated modulation of the Notch signaling pathway is linked to altered radiotherapy responsiveness in tongue squamous cell carcinoma [84]. Moreover, current evidence indicates that the lncRNA *HOTAIR* functions as a key regulator of anticancer therapy resistance by integrating epigenetic and post-transcriptional mechanisms that enhance tumor cell survival and plasticity, supporting its potential utility as both a predictive biomarker and a therapeutic target [86]. These studies consistently associate *HOTAIR* expression with alterations in programmed cell-death pathways, including suppression of apoptosis and dysregulation of autophagy-related signaling, thereby contributing to chemoresistance in cisplatin-treated OSCC cells [87,88]. In summary, the available evidence suggests that dysregulated lncRNA and circRNA regulatory networks in OSCC intersect with epigenetic regulators, stemness-associated pathways, metabolic adaptability, and therapeutic response mechanisms, underscoring their relevance as mechanistically informative biomarkers and as potential targets for strategies aimed at mitigating treatment resistance.

Although numerous studies have reported dysregulated microRNAs, lncRNAs, and circRNAs in OSCC, several limitations should be considered. Many proposed ncRNA regulatory networks are based on computational predictions or experimental models and lack validation in large clinical cohorts. Moreover, most studies focus on individual ncRNAs or specific pathways, while the broader interactions between ncRNAs and the tumor microenvironment remain poorly understood. In addition, challenges such as limited functional annotation, incomplete mechanistic validation, and inefficient delivery systems hinder the clinical translation of ncRNA-based biomarkers and therapies. Therefore, integrative multi-omics approaches and well-designed clinical studies are required to clarify the biological and translational significance of ncRNA networks in OSCC [141]. In addition, differences in sequencing platforms, experimental detection methods, and bioinformatic analysis pipelines may lead to variability in ncRNA profiling results across studies, as factors such as sample preparation, data normalization, and analytical workflows can influence the identification and interpretation of candidate non-coding RNA biomarkers [8]. Another limitation is that many proposed ncRNA regulatory networks are derived from bioinformatic predictions or correlation-based analyses, while experimental validation of these interactions remains incomplete. As a result, the functional relevance of some predicted ncRNA regulatory relationships in OSCC requires further mechanistic investigation [141,142]. Consequently, although non-coding RNA signatures show promise as diagnostic and therapeutic biomarkers in OSCC, large-scale multi-center validation studies and standardized analytical frameworks will be required to determine their true clinical applicability.

## 5. Dysregulated Signaling Pathways in OSCC

### 5.1. EGFR-Mediated PI3K/AKT/mTOR and MAPK Activation in OSCC: Implications for Tumor Progression and Therapeutic Resistance

*EGFR* overexpression and constitutive hyperactivation are defining molecular features of OSCC, with immunohistochemical and genomic studies consistently demonstrating elevated EGFR protein levels and gene copy number gains in a substantial proportion of tumors [89,90]. The EGFR-mediated PI3K/AKT/mTOR signaling axis represents one of the central oncogenic pathways in OSCC, regulating cell proliferation, metabolism, survival signaling, and therapeutic resistance. Aberrant EGFR signaling drives sustained activation of downstream PI3K/AKT/mTOR and MAPK cascades, thereby promoting uncontrolled proliferation, resistance to apoptosis, metabolic adaptation, and angiogenic signaling within the tumor microenvironment [91,92]. Activation of the PI3K/AKT axis enhances cell survival and anabolic metabolism, while MAPK pathway engagement supports mitogenic transcriptional programs and cell-cycle progression in OSCC cells [25,93]. In addition, aberrant EGFR activation promotes OSCC progression through the MAPK-dependent priming of the NLR family pyrin domain containing 3 (NLRP3) inflammasome and subsequent human leukocyte antigen-G (HLA-G) upregulation, contributing to an immunosuppressive tumor phenotype [94]. Clinically, EGFR expression has been associated with advanced tumor stage, locoregional invasion, and unfavorable OSCC patient outcomes [95]. Although EGFR-targeted agents such as cetuximab and small-molecule tyrosine kinase inhibitors are approved for HNSCC, therapeutic responses in OSCC remain heterogeneous and often transient [96]. This limited efficacy may reflect pathway redundancy, co-activation of parallel receptor tyrosine kinases, and adaptive re-engagement of downstream signaling components, including the PI3K/AKT/mTOR and MAPK pathways, which can occur even in the context of EGFR inhibition [94,97]. Overall, these observations underscore the central but context-dependent role of EGFR signaling in OSCC pathobiology and highlight the need for combinatorial or pathway-stratified therapeutic strategies.

### 5.2. Context-Dependent Reprogramming of TGF-β, Wnt, and Notch Signaling in OSCC: Implications for Tumor Progression and Precision Therapy

Transforming growth factor-β (TGF-β) signaling plays a context-dependent role in OSCC. During early tumorigenesis, TGF-β primarily functions as a tumor suppressor by inducing cell-cycle arrest, apoptosis, and cellular senescence. However, as tumor progression occurs, OSCC cells and the surrounding tumor microenvironment frequently reprogram TGF-β signaling toward oncogenic outputs. In advanced disease, enhanced TGF-β activity promotes EMT, tumor cell invasion, angiogenesis, and immune evasion, thereby facilitating metastatic dissemination and unfavorable clinical outcomes [98,99,100]. A previous study indicated that TGF-β facilitates OSCC progression by inducing CAF-like characteristics in normal fibroblasts, accompanied by augmented tumor growth and changes in angiogenic and matrix-remodeling pathways [101].

Wnt and Notch signaling pathways similarly exhibit complex and context-dependent functions in oral potentially malignant disorders and OSCC. These evolutionarily conserved pathways regulate cell fate determination and tissue homeostasis under physiological conditions; however, their dysregulation contributes to malignant transformation and tumor progression. Aberrant activation of Wnt/β-catenin signaling promotes EMT, metabolic reprogramming, and immune suppression within the tumor microenvironment, while altered Notch signaling has been associated with dedifferentiation, the acquisition of stem-like traits, and enhanced invasive capacity in OSCC [102,103,104,105,106]. Increasing evidence also indicates that cooperative interactions between Wnt and Notch signaling networks further reinforce oncogenic transcriptional programs and tumor cell plasticity.

Importantly, dysregulation of these signaling pathways has direct implications for therapeutic response in OSCC. TGF-β-driven EMT programs have been linked to resistance to radiotherapy and platinum-based chemotherapy through enhanced DNA damage tolerance, the suppression of apoptotic signaling, and increased cellular plasticity. TGF-β-driven EMT programs have been linked to resistance to radiotherapy and platinum-based chemotherapy through enhanced DNA damage tolerance and apoptosis suppression [143,144]. In parallel, the activation of Wnt/β-catenin signaling has been associated with immune exclusion phenotypes that limit tumor infiltration by cytotoxic lymphocytes and reduce responsiveness to immune checkpoint blockade. Activation of Wnt/β-catenin signaling has been associated with immune exclusion phenotypes that limit tumor infiltration by cytotoxic lymphocytes and reduce responsiveness to immune checkpoint blockade [145,146]. Persistent Notch signaling may also sustain therapy-resistant cancer stem-like cell populations capable of surviving cytotoxic stress and contributing to tumor recurrence [147].

These observations highlight the translational relevance of targeting these pathways in OSCC. Pharmacologic inhibitors of TGF-β, Wnt/β-catenin, and Notch signaling are currently being explored in both preclinical and clinical settings [148,149]. Combination strategies integrating pathway inhibitors with chemotherapy, radiotherapy, or PD-1/PD-L1-directed immunotherapy have also been proposed as promising approaches to overcome adaptive resistance mechanisms and enhance therapeutic efficacy in molecularly defined subsets of OSCC patients.

## 6. Immune Microenvironment and Immune Evasion

### 6.1. Immune Heterogeneity and Macrophage Regulation in OSCC

The OSCC immune landscape is characterized by complex infiltration patterns of T cells, B cells, macrophages, dendritic cells, and myeloid-derived suppressor cells (MDSCs) [107]. Although increased infiltration of cytotoxic CD8^+^ T lymphocytes is generally associated with effective antitumor immunity and improved clinical outcomes in many cancer types due to their capacity to directly recognize and kill malignant cells and produce effector cytokines, the overall impact of immune infiltrates is profoundly shaped by the broader tumor immune microenvironment [108]. Single-cell profiling identifies an expanded intratumoral population of CD4^+^ cytotoxic T lymphocytes exhibiting both effector and immunoregulatory programs, which engage closely with B-cell subsets and appear to contribute to the establishment of an immunosuppressive tumor milieu in OSCC [109]. A previous study provided a comprehensive synthesis of recent progress in leveraging natural killer (NK) cells as immunotherapeutic agents in OSCC, outlining their cytotoxic mechanisms, interactions within the tumor microenvironment, and novel platforms such as chimeric antigen receptor (CAR)-NK-based strategies and combinatorial checkpoint modulation [110]. Recent studies indicate that the OSCC tumor microenvironment contains a diverse mix of immune cell populations whose functional balance rather than sheer abundance critically determines antitumor immunity versus immune suppression [150,151,152]. While cytotoxic lymphocyte infiltration may confer benefit, emerging single-cell- and NK cell-focused research highlights that complex crosstalk among T cells, B cells, myeloid populations, and NK cells can instead promote an immunoregulatory milieu that shapes disease progression and informs novel immunotherapeutic strategies [110,153].

Macrophages constitute a key immunological component of the OSCC tumor microenvironment, where they orchestrate cancer cell proliferation, invasion, angiogenesis, and immune tolerance through complex bidirectional signaling with tumor and immune cells [111]. Increasing evidence indicates that distinct macrophage phenotypes exert divergent biological influences on OSCC behavior, highlighting their potential relevance as mechanistic biomarkers and therapeutic targets [112]. Conditioned media derived from OSCC cell lines promote the polarization of circulating monocytes into macrophages with an immunosuppressive CD25^+^CD163^+^CD206^+^ phenotype that attenuates T-cell activation, and this effect is attributable to soluble protein mediators rather than exosomal vesicles. Transcriptomic enrichment of cytokine–receptor signaling, including marked interleukin 2 receptor subunit alpha (IL2RA) upregulation, further supports OSCC-derived factors playing a role in instructing a tumor-permissive myeloid program within the microenvironment [113]. M1-polarized macrophages have been shown to enhance the proliferation, migration, invasion, and xenograft growth of OSCC cells, in part through growth differentiation factor 15 (GDF15)-mediated activation of erb-b2 receptor tyrosine kinase 2 (ErbB2) and downstream signaling pathways, including PI3K/AKT (see Section 5.1) and MAPK/ERK cascades [114]. In addition, loss of complement component 1 Q subcomponent-binding protein (C1QBP) expression in OSCC promotes a pro-tumorigenic microenvironment by facilitating M2 macrophage polarization through the activation of the tumor necrosis factor receptor associated factor 2 (TRAF2)-C-C motif chemokine ligand 2 (CCL2) signaling cascade [115]. Overall, accumulating evidence indicates that macrophages constitute a dynamic and functionally heterogeneous stromal population in OSCC, with tumor-derived factors shaping their polarization and signaling activity to foster a permissive microenvironment characterized by impaired antitumor immunity and augmented proliferative, invasive, and angiogenic capacities through mechanisms that include IL2RA-linked cytokine networks, GDF15-ErbB2-PI3K/AKT-MAPK axis activation, and TRAF2-CCL2-mediated M2 skewing [154,155,156]. These insights provide a rationale for therapeutic strategies aimed at modulating the tumor immune microenvironment, including immune checkpoint blockade and combination immunotherapies.

### 6.2. Clinical and Biological Significance of PD-1/PD-L1 Pathway Activation in OSCC

The programmed cell-death protein-1/programmed death-ligand 1 (PD-1/PD-L1) axis represents a key immune checkpoint pathway that enables tumor cells to evade immune surveillance in OSCC. Increasing evidence indicates that PD-L1 expression is frequently observed in OSCC and may influence tumor progression, immune regulation, and response to immunotherapy. Several studies have reported that PD-L1 expression increases along the oral carcinogenesis sequence, ranging from normal mucosa to oral epithelial dysplasia and ultimately invasive carcinoma, suggesting that immune checkpoint activation may occur early during tumor development [118,123]. In addition, PD-L1 expression has been detected in both tumor cells and tumor-infiltrating immune cells in a substantial proportion of OSCC cases.

From a clinicopathological perspective, PD-L1 expression has been associated with several indicators of aggressive tumor behavior. Elevated PD-L1 levels have been correlated with larger tumor size, greater invasion depth, lymphovascular invasion, and perineural invasion, supporting the potential role of PD-1/PD-L1 signaling in tumor progression and immune evasion [125]. Moreover, tumors arising in individuals without tobacco or alcohol exposure often demonstrate higher PD-L1 combined positive scores and an increased *PD-L1* gene copy number compared with those of exposure-related cases, suggesting that these tumors may represent a biologically distinct subset with unique immunological characteristics [119].

However, the prognostic significance of PD-L1 expression in OSCC remains controversial. While some studies have reported associations between high PD-L1 expression and unfavorable survival outcomes, other investigations have failed to demonstrate a consistent relationship between PD-L1 levels and overall survival [120,121]. These inconsistent findings likely reflect differences in patient populations, tumor biology, and study design across cohorts. Despite this variability in prognostic value, PD-L1 expression remains an important predictive biomarker for immune checkpoint inhibitor therapy. Clinical studies have shown that anti-PD-1 treatment can improve overall survival and enhance T-cell-mediated immune responses in OSCC patients, as reflected by increased interferon-γ production and the activation of CD4^+^ T-cell populations [124].

Mechanistically, PD-L1 expression in OSCC may be induced through both immune-mediated and oncogenic signaling pathways. Interferon-γ-mediated activation of the JAK/STAT signaling cascade represents a classical mechanism driving adaptive PD-L1 upregulation in response to antitumor immune activity. In addition, oncogenic signaling pathways, including EGFR-related cascades, have also been implicated in PD-L1 regulation, highlighting the complex interaction between tumor-intrinsic signaling networks and immune regulation within the tumor microenvironment [122].

Importantly, studies have struggled to interpret PD-L1 expression due to methodological heterogeneity in PD-L1 assessment. Differences in antibodies, staining platforms, and scoring systems, such as the tumor proportion score and combined positive score, may lead to variability in PD-L1 classification and contribute to inconsistent conclusions regarding its prognostic value. Greater standardization of PD-L1 evaluation methodologies will therefore be necessary to improve comparability among studies and to refine the clinical utility of PD-L1 as a biomarker for immunotherapy in OSCC. Collectively, current evidence suggests that although the prognostic significance of PD-L1 expression remains debated, the PD-1/PD-L1 axis represents a biologically and clinically important component of the OSCC immune microenvironment and continues to serve as a key biomarker guiding immunotherapeutic strategies.

Despite extensive investigation, the prognostic significance of PD-L1 expression in OSCC remains inconsistent across studies. While several reports have associated elevated PD-L1 levels with unfavorable survival outcomes, other studies have reported no significant correlation or even suggested potential associations with increased immune infiltration and improved clinical responses [157]. These discrepancies may arise from multiple methodological and biological factors, including differences in antibody clones, staining platforms, and scoring systems such as tumor proportion score (TPS) and combined positive score (CPS), as well as heterogeneity in patient populations and tumor subsites [158]. Moreover, PD-L1 expression is not a static tumor-intrinsic feature but is dynamically regulated by immune signaling within the tumor microenvironment. In particular, interferon-γ released by activated T cells can induce PD-L1 expression through the JAK–STAT signaling pathway, representing a form of adaptive immune resistance. This context-dependent regulation may complicate the interpretation of PD-L1 as a stable prognostic biomarker in OSCC [159]. Consequently, although PD-L1 remains clinically relevant as a predictive biomarker for immune checkpoint inhibitor therapy in head and neck cancers, its prognostic value in OSCC requires further clarification through standardized methodologies and prospective multi-center studies [160].

### 6.3. Central Roles of Cancer-Associated Fibroblasts in OSCC and HNSCC Progression and Therapy Resistance

Emerging evidence highlights that CAFs play a central role in HNSCC by interacting with tumor cells to promote growth, invasion, stemness, immune evasion, and therapy resistance, highlighting the importance of 3D tumor models and CAF-targeted therapeutic strategies [126]. CAF-mediated ECM remodeling creates physical barriers to immune infiltration, while CAF-derived cytokines skew immune polarization toward immunosuppressive phenotypes [127]. Zhang et al. proposed that CAFs in the OSCC microenvironment release exosomes lacking miR-148b-3p, which elevates ATPase copper transporting alpha (ATP7A) expression in tumor cells, reduces copper-induced cuproptosis, and ultimately drives tumor growth and metastasis [128]. CAFs markedly upregulate lysyl oxidase (LOX), which catalyzes collagen cross-linking and increases ECM stiffness, thereby activating focal adhesion kinase-dependent signaling to facilitate tumor cell proliferation, invasion, and EMT, highlighting LOX-mediated biomechanical remodeling as a critical determinant of malignant progression in OSCC [129]. Zhang et al. demonstrated that CAFs secrete C-X-C motif chemokine ligand 1, which suppresses differentiated embryo-chondrocyte expressed gene 2 expression in OSCC cells, driving dormant, cisplatin-resistant tumor cells to re-enter proliferation and promoting recurrence [130]. In a systematic review and meta-analysis of 11 studies including 1040 OSCC patients, the elevated cancer-associated fibroblast density, identified using α-smooth muscle actin, was generally associated with a significantly higher risk of mortality, indicating its potential value as an adverse prognostic biomarker despite variation across demographic subgroups [131]. Interestingly, Yamamoto et al. developed a 3D oral cancer model incorporating patient-derived CAFs to evaluate radiation responses and demonstrated that boron neutron capture therapy effectively reduced cancer invasion and selectively eliminated CAFs while sparing normal fibroblasts [132]. A previous study evaluated the distribution of CAFs in OSCC, histologically normal mucosa adjacent to OSCC, and verrucous carcinoma, demonstrating a stepwise increase in CAF frequency from adjacent mucosa to verrucous carcinoma and peaking in OSCC, which is consistent with progressively heightened biological aggressiveness [133]. Collectively, these observations underscore the pivotal roles of CAFs as key organizers of the OSCC tumor microenvironment and as clinically meaningful prognostic indicators, thereby reinforcing the importance of incorporating CAFs into advanced experimental models and supporting the rationale for developing targeted therapeutic strategies aimed at modulating CAF-mediated tumor-stroma interactions to improve clinical outcomes.

Despite growing recognition of the importance of cancer-associated fibroblasts in OSCC progression, several unresolved issues remain. One major challenge is the substantial phenotypic heterogeneity of CAF populations within the tumor microenvironment. Commonly used markers, including α-SMA, fibroblast activation protein (FAP), PDGFRβ, and vimentin, are not entirely specific and may label overlapping stromal cell populations, thereby complicating the interpretation of CAF-related findings across studies [161]. Furthermore, different CAF subpopulations may exert distinct biological functions, ranging from tumor-promoting activities such as extracellular matrix remodeling, immune suppression, and therapy resistance to potentially tumor-restraining roles in specific contexts [162]. These functional differences are not fully captured by conventional bulk analyses. Recent single-cell RNA sequencing and spatial transcriptomic analyses of HNSCC, including OSCC, have revealed substantial stromal heterogeneity and identified multiple transcriptionally distinct cancer-associated fibroblast subpopulations with diverse functional states and spatial distributions within the tumor microenvironment [163]. However, the functional significance and therapeutic relevance of these CAF subtypes remain incompletely understood and require further investigation.

## 7. Metabolic and Redox Reprogramming in OSCC: Mechanisms, Diagnostic Potential, and Therapeutic Implications

Metabolic reprogramming is increasingly recognized as a fundamental hallmark of OSCC, enabling tumor cells to sustain rapid proliferation, adapt to microenvironmental stress, and evade therapeutic pressure. Rather than representing isolated metabolic alterations, these changes involve the coordinated remodeling of glucose metabolism, lipid biosynthesis, and redox homeostasis, which collectively support tumor progression and treatment resistance within the tumor microenvironment [134].

One of the most prominent metabolic features of OSCC is enhanced glucose metabolism. Increased glucose uptake and glycolytic flux provide both energy and biosynthetic intermediates required for tumor growth and invasion. Several studies have reported that metabolic adaptations at the invasive tumor margin are associated with increased expression of glucose transporters and glycolysis-related enzymes. For example, reduced expression of Ras-related glycolysis inhibitor and calcium channel regulator (RRAD) has been linked to increased glucose metabolism and the upregulation of glucose transporter 3 (GLUT3), thereby facilitating metabolic adaptation in OSCC cells [136]. These findings support the concept that altered glucose metabolism contributes not only to tumor proliferation but also to spatial metabolic heterogeneity within the tumor microenvironment.

In addition to glucose metabolism, lipid metabolic reprogramming has emerged as another important metabolic characteristic of OSCC. Lipid metabolism supports membrane biosynthesis, signal transduction, and energy storage, all of which are essential for tumor cell survival and migration. Metabolomic analyses have revealed dysregulation of glycerophospholipid and sphingolipid pathways in OSCC, reflecting extensive lipid remodeling during tumor progression [135]. These alterations have also generated interest in lipid-based biomarkers, as metabolomic profiling studies have identified multi-lipid diagnostic models capable of distinguishing OSCC tissues from normal counterparts with high accuracy [137]. Such findings highlight the potential utility of lipid metabolic signatures as diagnostic tools for early detection and disease monitoring.

Beyond metabolic substrate utilization, redox regulation represents another key adaptive mechanism in OSCC. Tumor cells must maintain a delicate balance between reactive oxygen species (ROS) production and antioxidant defense systems in order to sustain proliferation while avoiding excessive oxidative damage. Alterations in ROS signaling have been linked to treatment resistance in several cancers, including OSCC. For example, increased expression of transcription factors such as forkhead box M1 (FOXM1) has been associated with radioresistant phenotypes by attenuating radiation-induced ROS accumulation and DNA damage responses [139]. These adaptive redox mechanisms enable tumor cells to tolerate oxidative stress generated by therapeutic interventions.

The metabolic and redox alterations observed in OSCC also have important diagnostic and therapeutic implications. Metabolomic profiling approaches based on lipid or metabolic signatures may facilitate the development of non-invasive biomarkers for early detection and risk stratification. From a therapeutic perspective, strategies targeting metabolic vulnerabilities or oxidative stress pathways are actively being explored. Several ROS-inducing approaches, including nanoengineered platforms and phytochemical-based pro-oxidant therapies, have demonstrated promising anticancer effects in preclinical models by selectively increasing oxidative stress in tumor cells [140]. In parallel, targeted agents such as the multi-target tyrosine kinase inhibitor anlotinib have been reported to induce oxidative stress and mitochondrial dysfunction in experimental OSCC systems, thereby promoting tumor cell apoptosis [138]. However, most ROS-based therapeutic strategies are primarily supported by preclinical evidence, and further clinical investigation is required to determine their translational potential.

Metabolic and redox reprogramming therefore represent interconnected regulatory processes that shape tumor progression, therapeutic resistance, and biomarker development in OSCC. Integrating metabolic profiling with emerging therapeutic strategies targeting metabolic and oxidative vulnerabilities may provide new opportunities for precision oncology approaches aimed at improving clinical outcomes in patients with OSCC. Targeting metabolic and redox vulnerabilities therefore represents a promising avenue for the development of precision therapeutic strategies in OSCC.

From a translational perspective, several molecular alterations observed in OSCC have potential clinical utility as biomarkers or therapeutic targets. For example, PD-L1 expression currently serves as a predictive biomarker for response to anti-PD-1 immune checkpoint inhibitors in recurrent or metastatic HNSCC. Immune checkpoint inhibitors such as pembrolizumab and nivolumab have been incorporated into clinical treatment algorithms for advanced HNSCC, highlighting the clinical relevance of biomarker-guided immunotherapy. Similarly, *EGFR* overexpression or amplification has provided the rationale for EGFR-targeted therapies such as cetuximab, although clinical responses remain heterogeneous. Alterations in the PI3K/AKT/mTOR pathway, including *PIK3CA* mutations, have also emerged as potential targets for pathway-specific inhibitors currently under clinical investigation. Several PI3K pathway inhibitors and combination strategies are currently being evaluated in early-phase clinical trials for HNSCC. Beyond tumor-intrinsic signaling, the tumor microenvironment represents an additional therapeutic dimension, with strategies targeting CAF-mediated stromal remodeling or immune-suppressive signaling networks being actively explored. In summary, these advances illustrate how molecular characterization of OSCC may support biomarker-guided patient stratification, inform therapeutic selection, and facilitate the development of rational combination treatment strategies.

Despite increasing interest in targeting tumor metabolism as a therapeutic strategy, several translational challenges remain. Cancer metabolic pathways exhibit substantial plasticity, allowing tumor cells to dynamically reprogram their metabolic networks and exploit alternative metabolic routes in response to therapeutic inhibition or microenvironmental stress [164]. Furthermore, metabolic interactions between tumor cells and stromal or immune compartments form a complex regulatory network within the tumor microenvironment, where metabolic competition and metabolite-mediated signaling can modulate immune cell activity and contribute to variability in therapeutic responses [165]. Another concern is that many metabolic pathways targeted in cancer therapy are also fundamental to normal cellular energy homeostasis, raising the possibility that metabolic inhibitors may disrupt physiological metabolic processes and lead to unintended toxicity in normal tissues [166]. Therefore, although metabolic targeting represents a promising therapeutic avenue in OSCC, further studies integrating metabolomics, spatial profiling, and clinical trials will be required to determine whether metabolic vulnerabilities can be effectively exploited for therapeutic benefit.

From a translational perspective, increasing understanding of OSCC molecular heterogeneity has opened opportunities for biomarker-guided therapeutic strategies. Immune checkpoint inhibitors targeting the PD-1/PD-L1 axis have demonstrated clinical benefit in recurrent or metastatic head and neck squamous cell carcinoma, highlighting the importance of immune landscape characterization for patient selection [157,167]. In parallel, aberrations in oncogenic pathways such as EGFR and PI3K/AKT/mTOR provide the rationale for targeted therapeutic strategies, although clinical responses remain heterogeneous [168,169]. Moreover, emerging evidence suggests that therapeutic approaches targeting tumor microenvironment components, including cancer-associated fibroblast-mediated stromal remodeling and immune suppression, may enhance treatment efficacy and overcome therapeutic resistance [170,171]. Integrating molecular biomarkers with multi-omics profiling and clinical stratification may therefore facilitate the development of more effective precision oncology strategies for OSCC. These findings highlight the importance of integrating molecular profiling with clinical decision-making in precision oncology.

## 8. Critical Evaluation and Translational Challenges

Although substantial progress has been made in understanding the molecular and microenvironmental basis of OSCC, several important limitations should be considered when interpreting the current body of evidence. In particular, the strength of the evidence supporting many proposed mechanisms varies considerably across studies.

First, a substantial proportion of mechanistic insights have been derived from in vitro experiments or preclinical animal models, which may not fully recapitulate the complexity and heterogeneity of human tumors. Consequently, the translational relevance of some proposed targets remains uncertain. Second, conflicting observations have been reported for several biomarkers, particularly immune checkpoint molecules such as PD-L1, whose prognostic value varies across cohorts and study designs. These inconsistencies may reflect differences in patient populations, analytical platforms, antibody selection, and scoring systems used for biomarker assessment. Third, many studies on signaling pathways or non-coding RNA networks rely on limited sample sizes or single-institution cohorts, which may introduce bias and reduce generalizability. In addition, experimental studies often focus on isolated pathways, whereas tumor behavior in vivo is shaped by complex interactions among genomic alterations, stromal components, and immune regulation. Finally, although multiple therapeutic strategies have been proposed based on emerging molecular insights, their clinical feasibility remains uncertain, and the clinical feasibility of translating these findings into effective therapies remains an ongoing challenge. Most candidate strategies are currently supported primarily by preclinical data, and robust clinical trials are required to determine their safety, efficacy, and patient selection criteria.

Addressing these challenges will require integrative multi-omics studies, standardized biomarker evaluation methods, and prospective clinical validation, which together may facilitate the translation of emerging molecular insights into clinically actionable strategies for OSCC.

## 9. Discussion

OSCC development is driven by complex interactions between tumor-intrinsic molecular alterations and the surrounding tumor microenvironment. Genomic and epigenetic changes can activate oncogenic signaling pathways that promote tumor proliferation, survival, and metabolic reprogramming. These tumor-intrinsic processes simultaneously reshape the tumor microenvironment by influencing immune cell infiltration, CAF activation, and extracellular matrix remodeling. In turn, stromal and immune components provide reciprocal signals that further modulate tumor signaling networks, metabolic adaptation, and therapeutic responses. Therefore, OSCC progression should be viewed as a dynamic and interconnected system in which genomic alterations, signaling pathways, stromal biology, immune regulation, and metabolic reprogramming collectively contribute to tumor heterogeneity and disease progression. This systems-level perspective provides an integrative framework linking tumor-intrinsic alterations with microenvironmental processes, which may help explain the biological heterogeneity and therapeutic resistance observed in OSCC.

Distinguishing established knowledge from unresolved questions will be critical for guiding future research priorities in OSCC. While substantial progress has been made in understanding the molecular mechanisms underlying OSCC, several aspects remain incompletely resolved. Established evidence indicates that genomic alterations, oncogenic signaling pathways, and tumor microenvironment interactions play central roles in OSCC development and progression. However, important controversies persist regarding the prognostic value of certain biomarkers, the functional heterogeneity of stromal components such as cancer-associated fibroblasts, and the clinical applicability of emerging molecular targets. Future studies integrating multi-omics profiling, spatial transcriptomics, and large-scale clinical validation will be essential to clarify these unresolved questions and facilitate the translation of molecular discoveries into precision therapeutic strategies for OSCC.

For PD-L1 prognostic controversy, despite extensive investigation, the prognostic significance of PD-L1 expression in OSCC remains controversial. Several studies have reported associations between high PD-L1 expression and unfavorable survival outcomes, whereas others have failed to demonstrate a consistent prognostic relationship. These discrepancies may reflect differences in patient cohorts, tumor subsites, antibody platforms, scoring systems such as TPS or CPS, and the dynamic regulation of PD-L1 by inflammatory signaling. Consequently, further standardized and prospective studies are required to clarify the clinical value of PD-L1 as a prognostic biomarker in OSCC. On the other hand, for CAF marker variability, although cancer-associated fibroblasts are widely recognized as key regulators of the OSCC tumor microenvironment, their phenotypic heterogeneity remains a major challenge. Commonly used markers such as α-SMA, FAP, PDGFRβ, and vimentin are not entirely specific and may label overlapping stromal cell populations. This variability complicates the interpretation of CAF-related findings and may partly explain inconsistencies across studies. Future investigations using single-cell and spatial transcriptomic approaches may help define functionally distinct CAF subpopulations and clarify their roles in tumor progression and therapeutic resistance. In this study, the limitations of non-coding RNA studies, in which numerous studies have identified dysregulated microRNAs, lncRNAs, and circRNAs in OSCC, should be acknowledged. Many existing reports rely on relatively small sample sizes, single-center cohorts, or in vitro experimental systems, which may limit the reproducibility and clinical applicability of the findings. In addition, differences in detection technologies, analytical pipelines, and data normalization strategies can contribute to inconsistent biomarker identification across studies. Therefore, larger multi-center investigations and standardized methodological approaches will be essential to validate the diagnostic and therapeutic relevance of non-coding RNA networks in OSCC. In discussion with metabolic targeting translational challenges, targeting tumor metabolism has emerged as a promising therapeutic strategy in OSCC; however, several translational challenges remain. Metabolic pathways are highly dynamic and often exhibit redundancy, allowing tumor cells to compensate for targeted metabolic inhibition. Furthermore, metabolic heterogeneity within the tumor microenvironment and potential toxicity to normal tissues complicate clinical application. Future studies integrating metabolomics, spatial profiling, and clinical trials will be required to determine whether metabolic vulnerabilities can be effectively exploited for therapeutic benefit.

Despite substantial advances in understanding OSCC biology, important knowledge gaps remain. Many proposed molecular mechanisms are primarily supported by preclinical evidence, and their clinical relevance requires further validation. Moreover, tumor progression is driven by complex interactions between genomic alterations, stromal components, immune regulation, and metabolic adaptation, highlighting the need for integrative research strategies. Future studies combining multi-omics profiling, spatial analysis, and prospective clinical cohorts will be essential to translate emerging molecular insights into clinically actionable biomarkers and precision therapeutic approaches.

Specifically, our review differs from many earlier articles in three key aspects. First, instead of discussing genomic alterations, tumor microenvironment components, and metabolic adaptations as independent topics, we emphasize their dynamic and bidirectional interactions, highlighting how tumor-intrinsic molecular alterations interact with stromal and immune regulatory networks to drive OSCC progression. This systems-oriented perspective is introduced in the conceptual framework presented in the Introduction and illustrated in Figure 1, which integrates genomic drivers, epigenetic reprogramming, immune regulation, CAF-mediated remodeling, and metabolic adaptation into a unified model of OSCC progression. Second, the manuscript aims to organize the rapidly expanding OSCC literature into a coherent biological hierarchy, linking molecular alterations, including *TP53* and *PIK3CA*, and signaling pathways with downstream tumor microenvironment remodeling processes and potential translational implications, including biomarkers and precision therapeutic strategies. Third, the review emphasizes the interconnected nature of tumor-intrinsic signaling pathways and microenvironmental regulation, highlighting how these processes collectively contribute to tumor heterogeneity, immune evasion, and therapeutic resistance. By presenting these processes within a system-level framework, the review attempts to bridge traditionally separate research domains in OSCC biology.

## 10. Conclusions

Oral squamous cell carcinoma (OSCC) is a highly heterogeneous malignancy driven by complex interactions between tumor-intrinsic molecular alterations and the surrounding tumor microenvironment. Genomic and epigenetic dysregulation activate oncogenic signaling pathways that promote tumor proliferation, immune evasion, stromal remodeling, and metabolic adaptation. Increasing evidence suggests that these processes operate within an interconnected regulatory network rather than as independent biological events. Advances in multi-omics technologies, single-cell sequencing, and spatial transcriptomics provide unprecedented insights into cellular heterogeneity and the dynamic interactions among tumor cells, stromal components, and immune populations. Importantly, translating these molecular insights into clinical benefit will require improved biomarker-guided patient stratification and therapeutic strategies that target both tumor cells and their microenvironment. A deeper system-level understanding of OSCC biology may therefore facilitate the development of more effective precision oncology approaches and ultimately improve clinical outcomes for patients with this challenging disease.

Overall, these findings highlight that effective OSCC management will likely require integrated therapeutic strategies targeting both tumor-intrinsic oncogenic pathways and the surrounding stromal and immune compartments. Continued advances in multi-omics technologies, spatial profiling, and precision oncology approaches are expected to further refine risk stratification and facilitate the development of more effective, individualized treatment strategies for patients with OSCC. These interconnected molecular and microenvironmental processes are conceptually summarized in Figure 1, which illustrates the system-level framework of OSCC progression discussed in this review. Future studies integrating multi-omics profiling, spatial biology, and clinical stratification may further accelerate the development of precision therapeutic strategies for OSCC.

## Figures and Tables

**Table 1:** Integrated molecular and microenvironmental drivers of OSCC progression and their translational implications.

Domain	Key Molecules or Cells	Major Roles in OSCC	Representative References
Epidemiology and Risk Factors	Betel quid, tobacco, alcohol, HPV	Drive geographic heterogeneity, chronic inflammation, and carcinogenesis	[1,2,3,4]
Core Genomic Drivers	*TP53*, *CDKN2A*(p16), *NOTCH1*, *PIK3CA*, *HRAS*	Genomic instability, cell-cycle dysregulation, context-dependent differentiation, oncogenic signaling	[11,12,13,14,15,16,17,18,19,20,21,22,23,24,25,26,27,28,29,30,31,32,33,34]
Copy Number Alterations	*EGFR*, *CCND1*, *MYC*, *SOX2*	Promote proliferation, stemness, therapy resistance, and intratumoral heterogeneity	[35,36,37,38,39]
Intratumoral Heterogeneity	Clonal evolution, EMT-like states	Enables adaptive resistance and recurrence under therapeutic pressure	[40,41,42,43,44,45]
DNA Methylation	*CDH1*, *CDKN2A*, *RASSF1A*	Promoter hypermethylation silences tumor suppressors; global hypomethylation induces instability	[46,47,48,49,50,51,52,53]
Histone Modifiers	HDACs, *EZH2* (PRC2)	Chromatin compaction, transcriptional repression, aggressive phenotype	[54,55,56,57,58,59,60,61,62,63]
MicroRNAs (miRNAs)	miR-21, miR-31, miR-145, miR-196, miR-204	Regulate PI3K/AKT/mTOR, EMT, invasion, prognosis; diagnostic saliva/plasma markers	[64,65,66,67,68,69,70,71]
lncRNAs/circRNAs	*MALAT1*, *HOTAIR*	Act as ceRNA and epigenetic scaffolds; promote EMT and cisplatin resistance	[72,73,74,75,76,77,78,79,80,81,82,83,84,85,86,87,88]
EGFR-Driven Signaling	EGFR–PI3K/AKT/mTOR, MAPK	Sustains proliferation, metabolism, immune evasion; limited response to monotherapy	[89,90,91,92,93,94,95,96,97]
TGF-β/Wnt/Notch	TGF-β, β-catenin, NOTCH1	Stage-dependent switch from tumor suppression to EMT, invasion, immune escape	[98,99,100,101,102,103,104,105,106]
Immune Landscape	CD8^+^ T cells, CD4^+^ T cells, NK cells	Functional balance determines antitumor vs. immunosuppressive state	[107,108,109,110]
Macrophage Polarization	M1/M2 TAMs, GDF15, CCL2	Promote invasion, angiogenesis, immune suppression	[111,112,113,114,115]
Immune Checkpoints	PD-1/PD-L1	Progressive upregulation from dysplasia to OSCC; heterogeneous prognostic value	[116,117,118,119,120,121,122,123,124,125]
CAFs	α-SMA^+^ CAFs, LOX, CXCL1	ECM stiffening, immune exclusion, therapy resistance, recurrence	[126,127,128,129,130,131,132,133]
Metabolic Reprogramming	Glycolysis, lipid metabolism, GLUT3	Supports invasion, immune modulation, and resistance	[134,135,136,137]
Redox Regulation	Reactive oxygen species (ROS), NOX5, FOXM1	Determines radio- and chemoresistance; exploitable vulnerability	[138,139,140]

## Data Availability

No new data were created or analyzed in this study. Data sharing is not applicable to this article.

## Abbreviations

The following abbreviations are used in this manuscript:

AKT	Protein kinase B
CAF(s)	Cancer-associated fibroblast(s)
CAMKIV	Calcium/calmodulin dependent protein kinase IV
CAR	Chimeric antigen receptor
CCL2	C-C motif chemokine ligand 2
CCND1	Cyclin D1
CDH1	Cadherin 1
CDKN2A	Cyclin-dependent kinase inhibitor 2A
ceRNA	Competing endogenous RNA
circRNA	Circular RNA
CNA	Copy number alteration
CREB1	CAMP responsive element binding protein 1
CTLA-4	Cytotoxic T-lymphocyte–associated protein 4
DGKG	Diacylglycerol kinase γ
ECM	Extracellular matrix
EGFR	Epidermal growth factor receptor
EMT	Epithelial–mesenchymal transition
ERCC1	Excision repair cross-complementation group 1
ERK	Extracellular signal-regulated kinase
EZH2	Enhancer of zeste homolog 2
FAK	Focal adhesion kinase
FAP	Fibroblast activation protein
FOXM1	Forkhead box M1
GDF15	Growth differentiation factor 15
GLUT3	Glucose transporter 3
HDAC	Histone deacetylase
HIF	Hypoxia-inducible factor
HLA-G	Human leukocyte antigen-G
HNSCC	Head and neck squamous cell carcinoma
HPV	Human papillomavirus
HRAS	Harvey rat sarcoma viral oncogene homolog
IFN-γ	Interferon gamma
IL	Interleukin
IL2RA	Interleukin 2 receptor subunit alpha
lncRNA	Long non-coding RNA
LOX	Lysyl oxidase
MAPK	Mitogen-activated protein kinase
MALAT1	Metastasis-associated lung adenocarcinoma transcript 1
MDSC(s)	Myeloid-derived suppressor cell(s)
miRNA	MicroRNA
MMP	Matrix metalloproteinase
mTOR	Mechanistic target of rapamycin
MYC	MYC proto-oncogene, BHLH transcription factor
NK cell(s)	Natural killer cell(s)
NLRP3	NLR family pyrin domain containing 3
NOTCH1-Dll4	Notch1–Delta-like-4
NOX5	NADPH oxidase 5
OSCC	Oral squamous cell carcinoma
PD-1	Programmed cell death protein 1
PD-L1	Programmed death-ligand 1
PI3K	Phosphatidylinositol 3-kinase
PIK3CA	Phosphatidylinositol-4,5-bisphosphate 3-kinase catalytic subunit alpha
PRC2	Polycomb repressive complex 2
PTEN	Phosphatase and tensin homolog
RASSF1A	Ras association domain family member 1 isoform A
ROS	Reactive oxygen species
RRAD	Ras-related glycolysis inhibitor and calcium channel regulator
SOX2	SRY-box transcription factor 2
TCGA	The Cancer Genome Atlas
TGF-β	Transforming growth factor beta
TME	Tumor microenvironment
TNF-α	Tumor necrosis factor alpha
TP53	Tumor protein p53
Wnt	Wingless/integrated signaling pathway
